# Trends in cognitive function before and after stroke in China

**DOI:** 10.1186/s12916-023-02908-5

**Published:** 2023-06-06

**Authors:** Jianian Hua, Jianye Dong, Guo-Chong Chen, Yueping Shen

**Affiliations:** 1 Department of Epidemiology and Biostatistics, School of Public Health, Medical College of Soochow University, 199 Ren’ai Road, Suzhou, 215123 China; 2 Department of Neurology, The First Affiliated Hospital of Soochow University, Suzhou, Jiangsu China; 3 Department of Obstetrics and Gynecology, The First Affiliated Hospital of Soochow University, Suzhou, Jiangsu China; 4 Department of Nutrition and Food Hygiene, Suzhou Medical College of Soochow University, 199 Ren’ai Road, Suzhou, 215123 China

**Keywords:** Stroke, Cognitive function, Chinese population, Cohort study

## Abstract

**Background:**

While cognitive impairment after stroke is common, cognitive trends before stroke are poorly understood, especially among the Chinese population who have a relatively high stroke burden. We aimed to model the trajectories of cognitive function before and after new-onset stroke among Chinese.

**Methods:**

A total of 13,311 Chinese participants aged ≥ 45 years and without a history of stroke were assessed at baseline between June 2011 and March 2012 and in at least one cognitive test between 2013 (wave 2) and 2018 (wave 4). Cognitive function was assessed using a global cognition score, which included episodic memory, visuospatial abilities, and a 10-item Telephone Interview of Cognitive Status (TICS-10) test to reflect calculation, attention, and orientation abilities.

**Results:**

During the 7-year follow-up, 610 (4.6%) participants experienced a first stroke. Both stroke and non-stroke groups showed declined cognitive function during follow-up. After adjustment for covariates, there was no significant difference in pre-stroke cognitive trajectories between stroke patients and stroke-free participants. The stroke group showed an acute decline in episodic memory (− 0.123 SD), visuospatial abilities (− 0.169 SD), and global cognition (− 0.135 SD) after stroke onset. In the years following stroke, the decline rate of the TICS-10 test was higher than the rate before stroke (− 0.045 SD/year).

**Conclusions:**

Chinese stroke patients had not experienced steeper declines in cognition before stroke compared with stroke-free individuals. Incident stroke was associated with acute declines in global cognition, episodic memory, visuospatial abilities, and accelerated declines in calculation, attention, and orientation abilities.

**Supplementary Information:**

The online version contains supplementary material available at 10.1186/s12916-023-02908-5.

## Background

Dementia and cognitive impairment are common after stroke [1]. A meta-analysis conducted in 2021 reported that approximately 10–30% of patients had dementia after stroke [2]. The prevalence of post-stroke cognitive impairment (PSCI) has recently increased owing to population aging and a decline in post-stroke mortality [3–5]. Nonetheless, little is known about the cognitive scores before stroke and the temporal patterns of change in cognition after stroke. Elucidating the cognitive trends pre- and post-stroke would be helpful to determine the impact of acute stroke treatment, type, severity, and classification on cognitive function and to design health policy to manage the burden of PSCI [6]. Given that most stroke studies are hospital-based, trajectories of cognitive function before stroke remain unclear. Baseline cognition, an important risk factor, is impossible to be measured. Two methods could provide clues to pre-stroke cognition. Informant Questionnaire of Cognitive Decline in the Elderly (IQCODE) consists of a series of questions about how cognitive function changed over a 10-year period, and it is applied to determine whether a stroke patient had pre-stroke cognitive impairment. Moreover, by searching medical records, researchers could know whether a participant has previously been diagnosed with dementia [7, 8]. However, these data are converted into categorical variables, limiting the observation of trends before and after stroke.

Only a few studies have investigated pre- and post-stroke cognitive trends and showed conflicting results. Some reported that stroke patients had lower cognitive scores and a steeper cognitive decline before stroke [9, 10], while others did not find such differences [11]. The domain-specific cognitive decline after stroke also differed across studies. One study reported that memory function showed not only an immediate decrement in the short term after stroke, but also a steeper decline rate in the long term after stroke [12]. Another study reported that the decline rate of memory function did not accelerate after stroke [9]. To the best of our knowledge, cognitive trends before and after stroke have not been investigated in the Chinese population, among whom there are a large number of stroke patients such that stroke is the leading cause of death and disability. The prevalence of PSCI is reported to be higher in China than in developed countries [2, 13]. Cognitive reserve, often indexed by cognitive function, education, and occupation, provides a buffer against the dementia-related pathology [14]. The Chinese population has different educational and economic levels, contributing to different cognitive reserves. Patients who had lower cognitive scores 6–12 months after stroke were reported to have a higher risk of dementia during an average of 3.8-year-follow-up [15]. An Americans in Reasons for Geographic and Racial Differences in Stroke (REGARDS) study ascertained that stroke patients with lower education showed different patterns of cognitive change after stroke, which was reflected by a greater acute cognitive decline in executive function [16]. Therefore, we hypothesized that the Chinese population might exhibit different patterns of cognitive trends.

In 2021, the China Health and Retirement Longitudinal Study (CHARLS), a nationally representative database, released its results from the fourth survey, offering the opportunity to explore the cognitive trends across multiple surveys over time. This study aimed to determine the temporal pattern of trends in cognitive function before and after stroke among the Chinese population.

## Methods

### Data source and study population

CHARLS is a community-based and nationally representative cohort study in China and a sister study of the Health and Retirement Study (HRS). The baseline (wave 1) was conducted in 2011 and included 17,708 Chinese participants randomly selected from 150 country units distributed in 28 provinces of China, via multistage probability sampling. Details of the sampling procedure and a description of the CHARLS are available elsewhere [17]. Data from wave 1 (2011), wave 2 (2013), wave 3 (2015), and wave 4 (2018) were used in the current analysis.

**Fig. 1:**
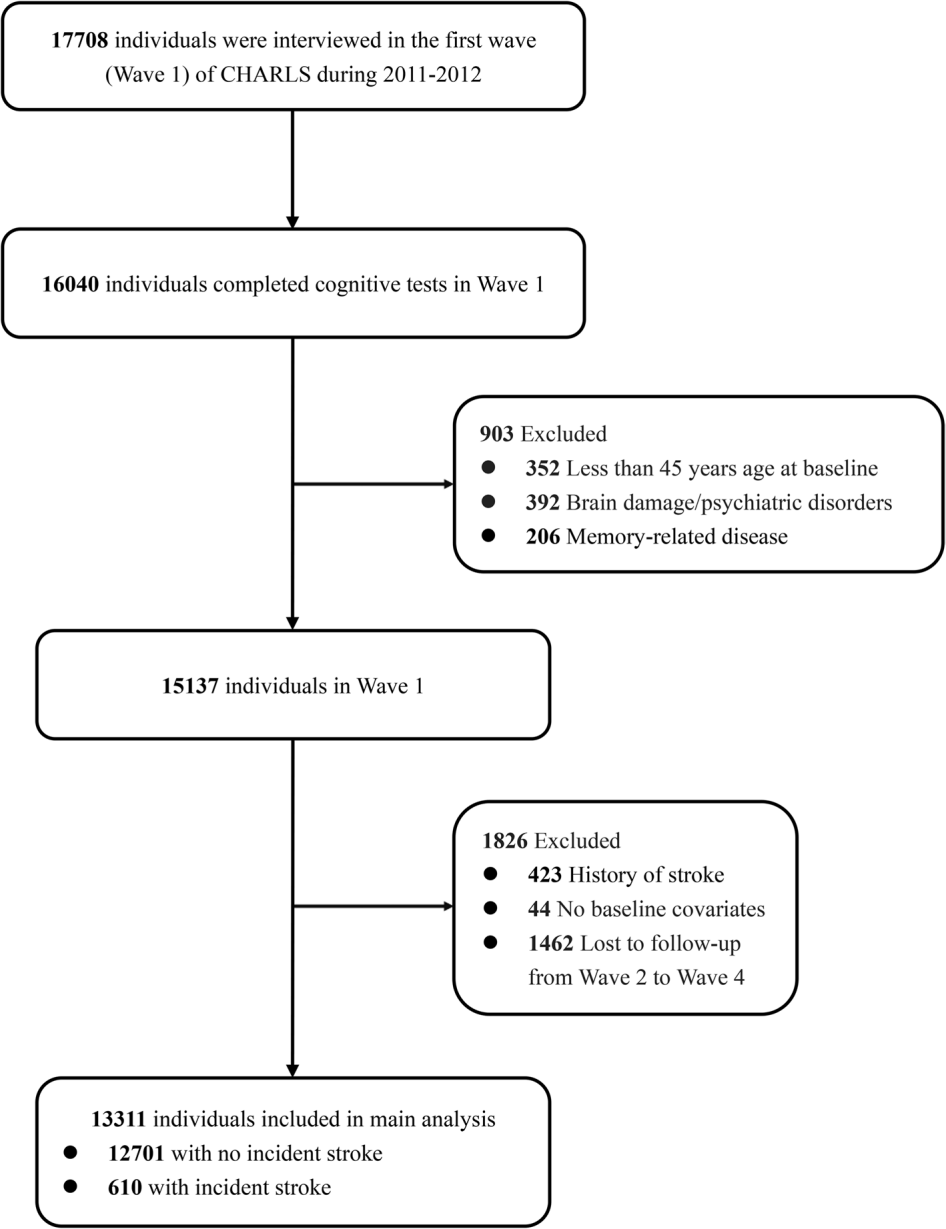
Participant selection flowchart

Figure 1 shows a flow chart of sample selection. The study population of CHARLS was middle-aged and older ($\ge$ 45 years) adults. Among the 16,040 individuals who completed cognitive tests at baseline, 903 participants aged $<$ 45 years at baseline (*n* = 352), with brain damage/psychiatric disorders (*n* = 392), or with memory-related diseases (*n* = 206) were excluded. The memory-related diseases were assessed by the question “Have you ever been diagnosed with memory-related diseases (like Alzheimer’s disease, atrophy, and Parkinson’s disease) by a doctor?” [17]. Of the 15,137 remaining participants, participants with a history of stroke at baseline (*n* = 423) or with missing baseline covariates (*n* = 44) were further excluded. One thousand four hundred sixty-two participants were excluded because they didn’t receive any cognitive tests from wave 2 to wave 4. Finally, 610 stroke survivors and 12,701 participants without incident stroke were included [12].

### Assessment of cognitive function and stroke

Based on the HRS, CHARLS designed a composite battery of cognitive tests. The reliability and validity of the assessments have been demonstrated in previous studies [18, 19]. The tests were administered face-to-face by well-trained interviewers, and they were conducted in four waves and included three domains. Immediate and delayed (5 min) recall tests of ten Chinese nouns were used to assess episodic memory. Each correct word was scored as 0.5 points, with total scores ranging from 0 to 10. Visuospatial abilities were assessed by asking the participants to redraw a figure. Success was scored as 1 point. The TICS-10 test asked participants to repeatedly subtract 7 from 100 and identify the date, season, and day of the week. This assessed the participants’ ability for calculation, attention, and orientation. The scores for the TICS-10 test ranged from 0 to 10.

*Z*-scores for each test were created using the mean and SD of the scores at the baseline. Global cognition *z*-scores were created by standardizing the sum of the three individual *z*-scores in the same manner [20].

Stroke was based on a time-updated self- or proxy- report of a doctor’s diagnosis (“Has a doctor ever told you that you had a stroke?”). No information on transient ischaemic attacks, stroke subtypes, or stroke severity was available. For some stroke participants, the diagnosis time was recorded through interviews. For others, the time was defined as the midpoint between the date of the last wave without stroke history and that of the first wave reporting a stroke history. In CHARLS, the time of stroke was only accurate to the year. By default, we set the measurement time of cognitive function at the mid-point of the year and the time of incident stroke at the beginning of the year. We then checked the questionnaire of each stroke participant to judge whether the stroke happened after the interview and then set the stroke time at the end of the year.

### Covariates

Covariates related to stroke and cognitive function, including age, sex, education, marital status, residential area, smoking, drinking, hypertension, dyslipidemia, diabetes, cancer, lung diseases, heart problems, depression, and the number of instrumental activities of daily living (IADLs) for which the participant needs help, were selected [12]. Education was classified as illiterate, primary school, middle school, and high school and above. Marital status was classified as married or other status. Lung diseases represented chronic lung diseases, such as chronic bronchitis and emphysema (excluding tumors, or cancer). Heart diseases included heart attack, coronary heart disease, angina, congestive heart failure, and other heart problems [17]. Depressive symptoms were measured using the 10-item Centre for Epidemiologic Studies Short Depression Scale, with depression defined as a score of ≥ 12 from a total score of 0 to 30.

### Statistical analysis

Baseline characteristics and baseline cognitive scores were compared between participants who did and did not experience a stroke during the follow-up and between those included in the analysis and those who were lost to follow-up. We constructed a linear mixed model to analyze the longitudinal dataset with repeated measurements. It is a two-level growth model. Time is our level-1 variable while participants are the level-2 units.

**Fig. 2:**
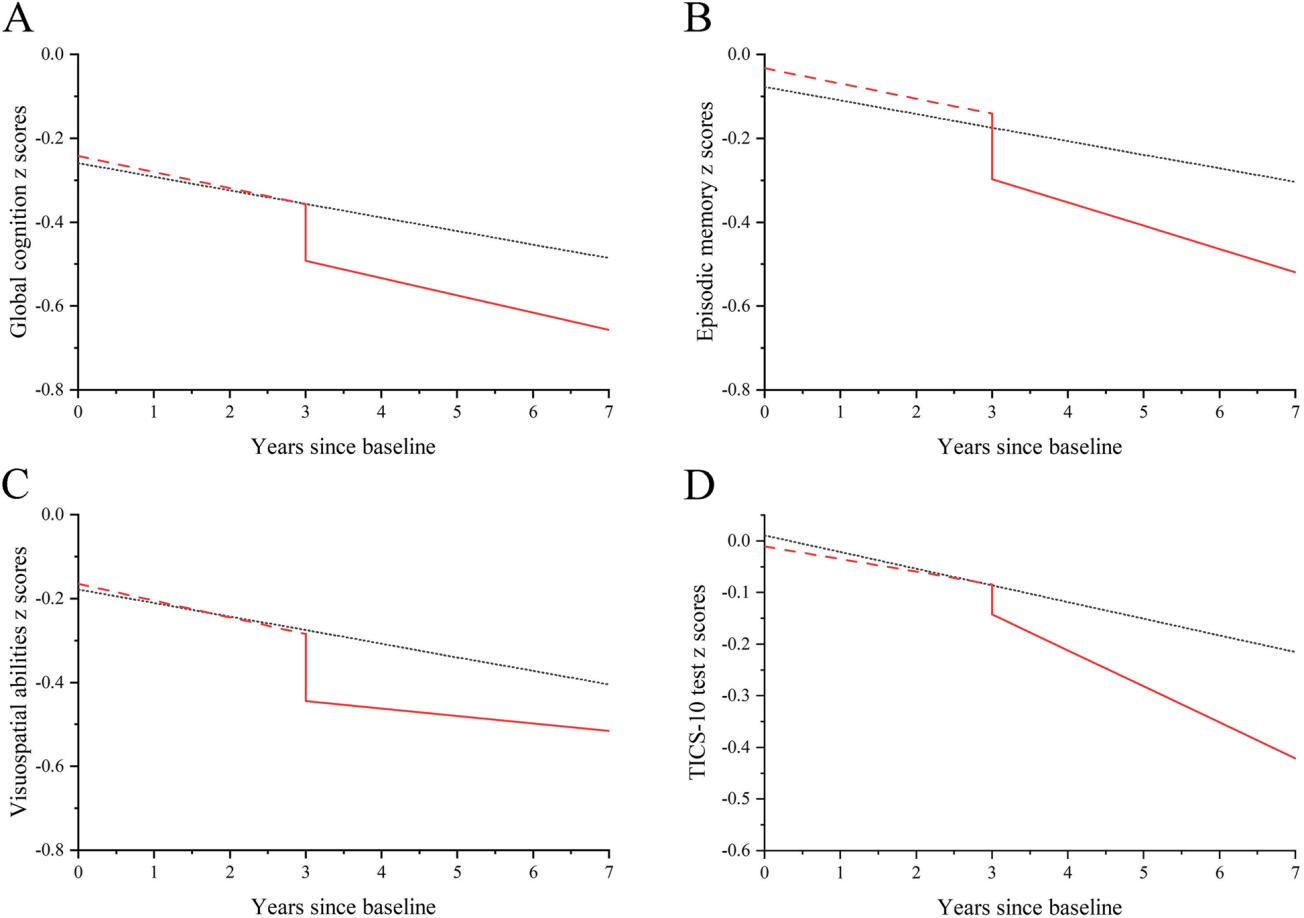
Predicted changes in cognitive *z* scores during the follow-up. The black dotted curves represent participants without incident stroke. The red dashed curves represent the pre-stroke cognitive trajectories among participants with incident stroke. The red solid curves represent the post-stroke trajectories. The predicted values of the cognitive score are calculated for a 70-year-old female. Her education level is primary school. She lives in a rural area and is married but without current smoking, current drinking, hypertension, dyslipidemia, diabetes, cancer, lung diseases, heart problems, or depression. Her IADL score is zero. She experienced one incident stroke at the end of the third year

In model A, we fitted fixed effects for intercept, incident stroke (yes or no), time (years since baseline), stroke*time interaction, stroke status (yes or no), and all the covariates. We fitted random effects for intercept and slope (time) to accommodate the correlation of cognitive measures within participants over time, to allow for the variation in average cognitive scores across participants, and to allow for the variation in average cognitive change rate across participants [21, 22]. The fixed effect parameter estimate for the variable incident stroke told us whether there were differences in baseline (time = 0) cognitive scores between the stroke group and the control group (without stroke). Similarly, the fixed effect parameter estimates for all covariates reflected the differences in baseline cognitive scores. The fixed effect of time showed the average cognitive change rate of the entire control group, while the stroke*time interaction evaluated the difference in cognitive change rates between participants with and without stroke. The “stroke status” was a time-varying dichotomous variable that changed from “0” to “1” at the time of stroke onset and reflected the acute cognitive decline in the short term after stroke. Model B included variates in Model A and further added a “stroke*stroke status*time-after-stroke” interaction. The time after stroke was fitted for both fixed and random effects. This interaction indicated changes in cognitive slope before and after stroke in stroke patients. Then, the stroke*time interaction reflected the difference between the average pre-stroke cognitive change rate for the stroke group and the average change rate from baseline to the end of follow-up for the control group. Figure S1 in the Additional file 1 shows the conceptual model [1, 23]. The visualization results of Model B are exhibited in Fig. 2. We chose to show the cognitive trajectories of a 70-year-old female with the most common baseline characteristics [12]. In Fig. 2, the random effects were set to zero.

In the sensitivity analysis, we included participants who had experienced a stroke before baseline. We restricted our subjects to participants (both with and without stroke) who underwent cognitive tests in all four waves. This tended to show the trends in relatively healthier participants [11]. We tested whether cognitive reserve (represented by education and baseline cognition), residential areas, age, sex, and medications after stroke modified the effect of stroke on the intercept and slope of cognitive trajectories using interaction terms. We further explored the association between baseline cognition and stroke risk. Kaplan–Meier curves, the Wilcoxon test, and the log-rank test were used to compare the cumulative risk of stroke over tertiles of baseline cognitive scores. Multivariate Cox proportional hazards regression was used to estimate hazard ratios and 95% confidence intervals.

All statistical analyses were performed using SAS version 9.4 (SAS Institute Inc., Cary, NC, USA). All *P* values were two-sided, with 0.05 being the threshold for statistical significance.

## Results

Among the 13,311 participants who were included in the main analysis, 610 (4.6%) experienced a first stroke during the 7-year follow-up period. The age-standardized rate of incident stroke is shown in Additional file 1: Table S1 [24]. Baseline characteristics according to stroke occurrence are shown in Table 1. The mean ± age of individuals without incident stroke was 58.6 ± 9.2 years. Moreover, 47.3% of them were male, 66.4% had not finished middle school, and 78.8% lived in rural areas. Compared with the non-stroke group, the stroke group was older and included a higher number of IADLs; higher percentages of being unmarried, smoking, drinking, hypertension, dyslipidemia, diabetes, lung disease, heart problems, and depression. Meanwhile, there was no between-group difference in sex proportions. Compared with without-stroke participants, stroke participants had lower baseline cognitive scores in all domains. However, after adjusting for age and sex, the difference turned out to be insignificant.

**Table 1:** Baseline participant characteristics by occurrence of incident stroke

	No incident stroke (*n* = 12,701)	Incident stroke (*n* = 610)	*P* value^*^	Adjusted *P* value^†^
Continuous variables, mean (SD)				
Age	58.6 (9.2)	61.0 (8.8)	< 0.001	-
Number of IADLs	0.2 (0.6)	0.3 (0.8)	< 0.001	< 0.001
Episodic memory scores	3.3 (1.9)	3.1 (1.8)	< 0.001	0.452
Visuospatial ability scores	0.6 (0.5)	0.6 (0.5)	0.003	0.054
TICS-10 test scores	6.6 (2.8)	6.3 (2.9)	< 0.001	0.109
Categorical variables, *n* (%)				
Males	5925 (47.3)	290 (48.3)	0.335	-
Education			< 0.001	0.247
Illiterate	3293 (26.3)	173 (28.8)		
Primary school	5026 (40.1)	258 (42.9)		
Middle school	2665 (21.3)	104 (17.3)		
High school and above	1556 (12.4)	66 (11.0)		
Marital status			< 0.001	< 0.001
Married	11,156 (89.0)	520 (86.5)		
Other status	1384 (11.0)	81 (13.5)		
Residential area			0.285	0.002
Urban	2660 (21.2)	122 (20.3)		
Rural	9880 (78.8)	479 (79.7)		
Current smoking	4861 (38.8)	254 (42.3)	< 0.001	< 0.001
Current drinking	3201 (25.2)	165 (27.5)	0.034	< 0.001
Hypertension	2731 (21.8)	245 (40.8)	< 0.001	< 0.001
Dyslipidemia	1041 (8.30)	89 (14.8)	< 0.001	< 0.001
Diabetes	633 (5.1)	51 (8.5)	< 0.001	< 0.001
Cancer	111 (0.9)	3 (0.5)	0.046	0.079
Lung diseases	1228 (9.8)	68 (11.3)	0.015	< 0.001
Heart problems	1384 (11.4)	107 (17.8)	< 0.001	< 0.001
Depression	2579 (20.6)	165 (27.5)	< 0.001	< 0.001

*TICS-10*, 10-item Telephone Interview of Cognitive Status

^*^Calculated using ANOVA for continuous covariates and *χ* ^2^ test for categorical covariates

^†^Calculated using generalized linear models for continuous covariates and logistic regression for categorical covariates after adjustment for baseline age and sex

From waves 1 to 4, the available cognition measurements were 12,701, 11,386, 11,023, and 8792 in the control group, and 610, 557, 550, and 458 in the stroke group, respectively (Additional file 1: Table S2). We excluded 1462 participants due to loss to follow-up. The nonresponders were older; had lower cognitive scores and a higher number of IADLs; had a higher percentage of unmarried, living in urban areas, hypertension, diabetes mellitus, cancer, chronic lung disease, and heart problems (Additional file 1: Table S3).

The linear mixed models reflected the following trajectories of cognitive function: (1) the predicted baseline score of the control group (intercept and covariates), (2) the average change rate of the non-stroke group (slope), (3) the difference in baseline scores between stroke group and control group (difference in baseline), (4) the difference in decline rate in the stroke group during the pre-stroke period compared to the control group (difference in slope before stroke), (5) the acute cognitive change after stroke among the stroke group, and (6) how much the decline rate change after stroke among the stroke group in the long term. As shown in Table 2, there was no between-group difference in the baseline cognitive scores. The non-stroke group showed a continuous decline in cognitive scores in all cognitive domains. Similarly, the cognitive function of the stroke group showed continuously declined cognitive function before stroke onset. The rates of decline were similar between the two groups. Therefore, we did not observe pre-stroke cognitive disadvantages in people with stroke compared to people without stroke.

**Table 2:** Changes in cognitive function among all participants over time

	Global cognition		Episodic memory		Visuospatial ability		TICS-10 test	
	Model A	Model B	Model A	Model B	Model A	Model B	Model A	Model B
	*β* (95% CI)	*β* (95% CI)	*β* (95% CI)	*β* (95% CI)	*β* (95% CI)	*β* (95% CI)	*β* (95% CI)	*β* (95% CI)
Variables								
Baseline age	− 0.018 (− 0.020, − 0.017)	− 0.018 (− 0.020, − 0.017)	− 0.021 (− 0.023, − 0.020)	− 0.021 (− 0.023, − 0.020)	− 0.012 (− 0.013, − 0.011)	− 0.012 (− 0.013, − 0.011)	− 0.009 (− 0.010, − 0.007)	− 0.009 (− 0.010, − 0.007)
Intercept of without-stroke group	0.221 (0.114, 0.328)	0.221 (0.115, 0.328)	0.540 (0.435, 0.646)	0.541 (0.435, 0.647)	0.213 (0.107, 0.318)	0.212 (0.107, 0.318)	0.174 (0.072, 0.277)	0.175 (0.073, 0.278)
Difference in baseline^a^	0.017 (− 0.048, 0.082)	0.017 (− 0.050, 0.085)	0.059 (− 0.012, 0.130)	0.045 (− 0.030, 0.119)	− 0.002 (− 0.077, 0.074)	0.014 (− 0.065, 0.093)	0.012 (− 0.050, 0.074)	− 0.021 (− 0.087, 0.044)
Slope of without-stroke group	− 0.032 (− 0.035, − 0.030)	− 0.032 (− 0.035, − 0.030)	− 0.013 (− 0.016, − 0.010)	-0.013 (− 0.016, − 0.010)	− 0.037 (− 0.041, − 0.034)	− 0.037 (− 0.041, − 0.034)	− 0.050 (− 0.052, − 0.048)	− 0.050 (− 0.052, − 0.048)
Difference in slope before stroke^b^	− 0.005 (− 0.023, 0.011)	− 0.006 (− 0.026, 0.014)	− 0.010 (− 0.030, 0.009)	− 0.004 (− 0.026, 0.018)	− 0.001 (− 0.023, 0.022)	− 0.008 (− 0.032, 0.017)	− 0.008 (− 0.025, 0.008)	0.008 (− 0.011, 0.027)
Acute change after stroke^c^	− 0.138 (− 0.227, − 0.049)	− 0.135 (− 0.228, − 0.042)	− 0.139 (− 0.239, − 0.039)	− 0.123 (− 0.227, − 0.019)	− 0.143 (− 0.258, − 0.029)	− 0.169 (− 0.292, − 0.047)	− 0.086 (− 0.176, 0.004)	− 0.056 (− 0.148, 0.035)
Changes in slope after stroke	None	− 0.003 (− 0.035, 0.029)	None	− 0.019 (− 0.052, 0.014)	None	0.022 (− 0.017, 0.062)	None	− 0.045 (− 0.073, − 0.017)
Log likelihood	− 47,397.5	− 47,396.3	− 57,312.6	− 57,310.1	− 58,379.4	− 58,377.1	− 53,235.9	− 53,227.9

All coefficients and confidence intervals are shown as *z* scores

Adjusted for baseline age (shown in line “Baseline age”), sex, education, marital status, residential area, current smoking, current drinking, hypertension, dyslipidemia, diabetes, cancer, lung diseases, heart problems, depression, and number of IADLs

*TICS-10*, 10-item Telephone Interview of Cognitive Status

^a^The difference in baseline cognitive scores between the stroke group and non-stroke group

^b^The difference in slope during the pre-stroke period in the stroke group compared to that in the non-stroke group during the entire follow-up period

^c^The amount of cognitive scores changed at the “stroke point” among the stroke group, measured as the first post-stroke value minus the last pre-stroke value

The acute cognitive decline after stroke is shown in Table 2 and by the vertical line in Fig. 2. Stroke survivors showed a significant acute decline in episodic memory (*β*, − 0.123 SD; 95% CI, − 0.227 to − 0.019; *P* = 0.021; Model B), visuospatial ability (*β*, − 0.169 SD; 95% CI, − 0.292 to − 0.047; *P* = 0.007; Model B), and global cognition (*β*, − 0.135 SD; 95% CI, − 0.228 to − 0.042; *P* = 0.004; Model B). The acute decline in episodic memory, visuospatial ability, and global cognition was equivalent to 9, 5, and 4 years of aging, respectively. However, there was no acute cognitive decline after incident stroke in TICS-10 test scores (*β*, − 0.056 SD; 95% CI, − 0.148 to − 0.035; *P* = 0.230; Model B).

In the years following stroke, all cognitive domains declined over time (Fig. 2). The TICS-10 test scores declined 0.045 SD/year faster than it did before stroke (*β*, − 0.045 SD; 95% CI, − 0.073 to − 0.017; *P* = 0.001; Model B). The accelerated decline per year was equivalent to 1 year of aging. However, compared with the pre-stroke rate, there was no accelerated decline in episodic memory, visuospatial ability, or global cognition among stroke survivors.

Sensitivity analysis of participants with baseline stroke (Additional file 1: Table S4) or of including participants who underwent cognitive tests in all four waves (Additional file 1: Table S5) showed similar trajectories before and after stroke. Residential areas did not significantly modify the effect of stroke on cognitive trends (Additional file 1: Table S6). Stroke participants with better education had more acute cognitive decline at the time of stroke and slower cognitive decline in the long time after stroke (Additional file 1: Table S7). Compared with stroke patients in the highest baseline cognitive tertile, those in the lowest tertile turned out to have lower baseline scores and faster cognitive decline in the long-term after stroke (Additional file 1: Table S8). Each 1-year increase in age did not significantly modify the cognitive slope after stroke (Additional file 1: Table S9). Compared with males, females showed a less acute cognitive decline in visuospatial ability (Additional file 1: Table S10). Those taking western stroke medicine after stroke showed a more acute cognitive decline in global cognition, visuospatial ability, and TICS-10 test, and slower cognitive decline in global cognition and visuospatial ability (Additional file 1: Table S11). Individuals with lower baseline scores were more likely to be attacked by stroke during the follow-up (Additional file 1: Figure S2). Nonetheless, after adjusting for covariates, only participants with the mid tertile of TICS-10 test scores had higher risks of stroke (adjusted HR, 1.252; 95% CI, 1.025, 1.530) during the follow-up, compared with participants with the highest tertile of TICS-10 test scores (Additional file 1: Table S12).

## Discussion

Evidence of cognitive trends before stroke is rare, especially among the Chinese population. In this national cohort of Chinese adults aged ≥ 45 years, there was no significant difference in baseline cognition and pre-stroke cognitive decline rate between participants with and without stroke. For episodic memory abilities, visuospatial abilities, and global cognition, stroke patients experienced an acute cognitive decline at the time of stroke and showed the same after-stroke decline rate as their pre-stroke rate. For the TICS-10 test, which reflected calculation, attention, and orientation abilities, stroke participants did not show an immediate cognitive decline at stroke onset but experienced a steeper cognitive decline in the long term after stroke.

A key difference between our study and previous findings lies in the pre-stroke cognitive scores. The study using English Longitudinal Study of Ageing (ELSA) [12], from the UK, reported that compared with stroke-free participants, stroke patients experienced faster cognitive decline in all domains before stroke. The domains included memory, verbal fluency, and temporal orientation. This might be because stroke patients had higher comorbidity of cardiovascular and cognitive risk factors during quite a significant time before stroke onset. These risk factors could accelerate cognitive decline even after they were adjusted in the multiple regression [25]. In the HRS Study [9, 26], which was conducted in America, the pre-stroke decline in memory function was steeper in stroke survivors and much steeper in stroke decedents than in stroke-free participants [9]. The study using ELSA did not consider the effect of fatal stroke [12], and it is unclear whether the results would change after subgroup analysis. A disadvantage of the CHARLS database was that it did not record the cause of death. Participants who died due to stroke were assigned to the control group. Thus, our study is more likely to reflect the situation among stroke survivors. From 2011 to 2018, the age-standardized incidence rate of stroke was 5.8/1,000 person-years in our dataset. The actual incidence rate was higher after considering fatal stroke. In 2013, a nationwide Chinese survey reported that the age-standardized incidence rate of stroke was 246.8/100,000 person-years among individuals aged ≥ 20 years [27]. Our results suggest that the incidence of stroke in China is increasing.

Another main finding of our study was the patterns of change in different cognitive domains after stroke. To our knowledge, this is the first study to report the trajectories of visuospatial abilities before and after stroke [9–12, 28]. With regard to memory abilities, we showed patterns different from those of other ethnicities. For the UK participants in ELSA [12] and REGARDS [11, 16], the memory function declined acutely at stroke onset and declined faster in the long term after stroke. However, the Americans in the HRS only experienced an acute cognitive decline immediately after stroke. The decline rate of their memory function remained similar after stroke [9, 26]. The usage of *z*-scores makes results across studies comparable. The acute cognitive decline in memory among the Chinese population (− 0.123 SD) was similar to that among the UK population (− 0.150 SD) and less than that among the American population in Epoch 3 of HRS (− 0.25 SD) [9, 12].

The acute cognitive decline could be explained by mechanisms of cognitive impairment in the acute and subacute stage of stroke, including infarction of the brain tissue, hypoperfusion, and “misery perfusion,” which is characterized by decreased cerebral blood flow and increased oxygen extraction fraction in areas distal to the lesions [29–31]. Mechanisms underlying long-term cognitive decline after stroke remain to be elucidated. Incident stroke can trigger or accelerate Alzheimer’s disease-related pathologies, such as amyloid deposition [32]. Meanwhile, neurodegeneration ahead of stroke could exacerbate brain injury after stroke [33, 34]. Stroke survivors had a higher prevalence of hypertension, diabetes, and atrial fibrillations. Vascular-related comorbidities and immune responses contribute to cognitive deficits after stroke [25, 35]. Cerebral small vessel disease, reflected by white matter hyperintensity, lacunes, perivascular space, and microbleeds, is associated with greater cognitive decline in general cognition [36, 37]. Recurrent stroke might increase the risk of dementia in the long term after stroke [1, 33]. In our study, the TICS-10 test did not show acute cognitive decline but accelerated decline. We are unable to figure out the reason for different cognitive patterns. A previous study reported different stroke-related lesions led to impairment in different cognitive domains. The kind of lesions could help explain the different results [7]. Recurrent stroke, either clinical or subclinical, and cerebral small vessel diseases, are associated with the long-term cognitive decline. Future studies could learn whether these associations were more pronounced in the TICS-10 test. Understanding the reason behind these findings may shed light on the treatments for stroke and provide effective interventions to reduce the cognitive burden after stroke.

The main advantage of this study is our population. To our best knowledge, this study is the first to report how the cognitive trajectories differed before and after incident stroke among Chinese participants. We adjusted for a number of covariates which were associated with cognition. Previous studies that focused on stroke survivors missed pre-stroke cognitive tests and might not be generalizable to the Chinese population. The mortality of stroke in China has been decreasing in recent years owing to improvements in therapy and treatment [38, 39], such as the growing use of recombinant tissue-type plasminogen activator(r-tPA) and standard therapy. Currently, China has over 300 comprehensive stroke centers and over 1000 tertiary stroke centers. In recent decades, a growing number of observational studies have reported the prevalence and types of cognitive or emotional dysfunction after stroke in developing countries [40]. In this regard, studies in China are dedicated not only to motor dysfunction, but also to complications such as cognitive decline. The cognitive trajectories revealed by our study highlight the need for improved cognitive assessments in stroke trials. Compared with previous studies, we also reported different patterns of cognitive decline in multi-domains [12, 28].

However, our study also has some limitations. First, medical records were lacking, leading to several incomplete data. We were unable to differentiate among stroke subtypes, including cerebral infarction, intracerebral hemorrhage, subarachnoid hemorrhage, or transient ischaemic attacks. Given that ischaemic stroke accounts for approximately 80% of stroke cases, our results are more likely to reflect conditions in ischaemic stroke survivors. Furthermore, self-reported or investigator-reported stroke status may introduce recall bias. An American study showed that self- and proxy-reported stroke history had a sensitivity of 74% and a specificity of 93% for detecting stroke [41]. Data on the validity of self-reported stroke among the Chinese population is lacking. Future studies could recruit doctors to re-evaluate the diagnosis. Insufficient data on stroke severity, location, or acute treatment prevented us from controlling for more factors [7]. Only 375 (61%) of our stroke participants reported taking western modern stroke medicine after a stroke. Others took Chinese medicine, receive physical therapy or acupuncture therapy, or even did not receive therapy. We encourage investigators of the cohort study to help their stroke participants receive therapy in the nearby stroke center. Second, our cohort did not record the cause of death. And thus, we were unable to identify stroke decedents who experienced a stroke and soon died during one wave. Participants who had a severe stroke or was aphasia after stroke were more likely to die or drop out and tended to have poorer health status at baseline. This might reduce our ability to detect the full effects of stroke on cognitive trends, especially trends before stroke. Future studies could record the reason why a participant does not finish the cognitive test (e.g., died or disabled due to stroke) so that the researchers could perform subgroup analysis. Third, although we controlled for many confounders, we cannot rule out the possibility of residual confounders such as atrial fibrillation, body mass index, air pollution, and apolipoprotein E genotype. Fourth, for most stroke patients, their cognitive scores declined in the acute phase of stroke and partly recovered 3–6 months after stroke. The recovery was not complete, since the dementia risk 6–12 months after stroke was still 2–5 times higher than that several years after stroke [23]. The acute cognitive decline in our models partly reflected this decline in the short term after stroke. However, due to limited time points for observation, we could not see the recovery process of cognitive function. We urge future cohort studies to additionally interview their participants 3, 6, and 12 months after the stroke event.

## Conclusions

Stroke would lead to acute cognitive decline in the short-term after stroke. Over the long period after stroke, patients would experience faster cognitive decline compared with the trends before stroke.

Furthermore, we reveal different patterns of post-stroke cognitive decline. Before stroke onset, trajectories of cognitive function among Chinese stroke patients were similar to those of stroke-free participants after adjusted for covariates. Incident stroke was associated with acute declines in global cognition, episodic memory, visuospatial abilities, and accelerated decline in orientation, attention, and calculation abilities.

## Supplementary Information

**Additional file 1: Supplemental Texts.** Ethics Approval. **Figure S1.** The conceptual model of our study. **Figure S2.** Kaplan-Meier curves for the cumulative risk of stroke in tertiles. **Table S1.** Age-standardised stroke incidence rates. **Table S2.** Number of available cognition measurements in each wave. **Table S3.** Comparison of baseline characteristics between participants includedand excluded due to loss to follow-up. **Table S4.** Adjusted changes in cognitive function over time: including 423 participants with a history of stroke at baseline. **Table S5.** Adjusted changes in cognitive function over time: only including participants received cognitive tests in all four waves. **Table S6.** Effect of living in urban areas on the effect of stroke on the following cognitive trajectories. **Table S7.** Effect of education on the effect of stroke on the following cognitive trajectories. **Table S8.** Effect of baseline cognition on the effect of stroke on the following cognitive trajectories. **Table S9.** Effect of 1-year increase in baseline age on the effect of stroke on the following cognitive trajectories. **Table S10.** Effect of female sex on the effect of stroke on the following cognitive trajectories. **Table S11.** Effect of western stroke medicine on the effect of stroke on the following cognitive trajectories. **Table S12.** Association between baseline cognitive function and new-onset stroke.

## Data Availability

Data used in this manuscript from the China Health and Retirement Longitudinal Study (CHARLS). We applied permission for data access (http://charls.pku.edu.cn/zh-CN) and obtained access to use it. Prof. Yaohui Zhao (National School of Development of Peking University), John Strauss (University of Southern California), and Gonghuan Yang (Chinese Center for Disease Control and Prevention) are the principal investigators. Requests to access these data can also be directed to Yueping Shen (shenyueping@suda.edu.cn).

## Abbreviations

CHARLS: China Health and Retirement Longitudinal Study
ELSA: English Longitudinal Study of Ageing
HRS: Health and Retirement Study
IADLs: Instrumental activities of daily living
IQCODE: Informant Questionnaire of Cognitive Decline in the Elderly
PSCI: Post-stroke cognitive impairment
REGARDS: Reasons for Geographic and Racial Differences in Stroke
TICS-10: 10-Item Telephone Interview of Cognitive Status
