# Supplementary material for: Trends in cognitive function before and after stroke in China
Source: BMC Med. 2023 Jun 6;21:204. doi: 10.1186/s12916-023-02908-5 (PMC10242976; doi:10.1186/s12916-023-02908-5)
Supplement: Supplementary file 1 — Additional file 1: Supplemental Texts. Ethics Approval. Figure S1. The conceptual model of our study. Figure S2. Kaplan-Meier curves for the cumulative risk of stroke in tertiles. Table S1. Age-standardised stroke incidence rates. Table S2. Number of available cognition measurements in each wave. Table S3. Comparison of baseline characteristics between participants includedand excluded due to loss to follow-up. Table S4. Adjusted changes in cognitive function over time: including 423 participants with a history of stroke at baseline. Table S5. Adjusted changes in cognitive function over time: only including participants received cognitive tests in all four waves. Table S6. Effect of living in urban areas on the effect of stroke on the following cognitive trajectories. Table S7. Effect of education on the effect of stroke on the following cognitive trajectories. Table S8. Effect of baseline cognition on the effect of stroke on the following cognitive trajectories. Table S9. Effect of 1-year increase in baseline age on the effect of stroke on the following cognitive trajectories. Table S10. Effect of female sex on the effect of stroke on the following cognitive trajectories. Table S11. Effect of western stroke medicine on the effect of stroke on the following cognitive trajectories. Table S12. Association between baseline cognitive function and new-onset stroke. [file 12916_2023_2908_MOESM1_ESM.docx]

# Additional file 1

# Supplemental Texts. Ethics approval

Ethical approval for all the CHARLS waves was granted from the Institutional Review Board at Peking University. The IRB approval number for the main household survey, including anthropometrics, is IRB00001052-11015; the IRB approval number for biomarker collection is IRB00001052-11014. During the fieldwork, each respondent who agreed to participate in the survey was asked to sign two copies of informed consent; one copy was kept in the CHARLS office, which was also scanned and saved in PDF format. Four separate consents were obtained: one for the main fieldwork, one for the non-blood biomarkers and one for the taking of the blood samples, and another for storage of blood for future analyses.

# Supplemental Figures

## Figure S1. The conceptual model of our study.


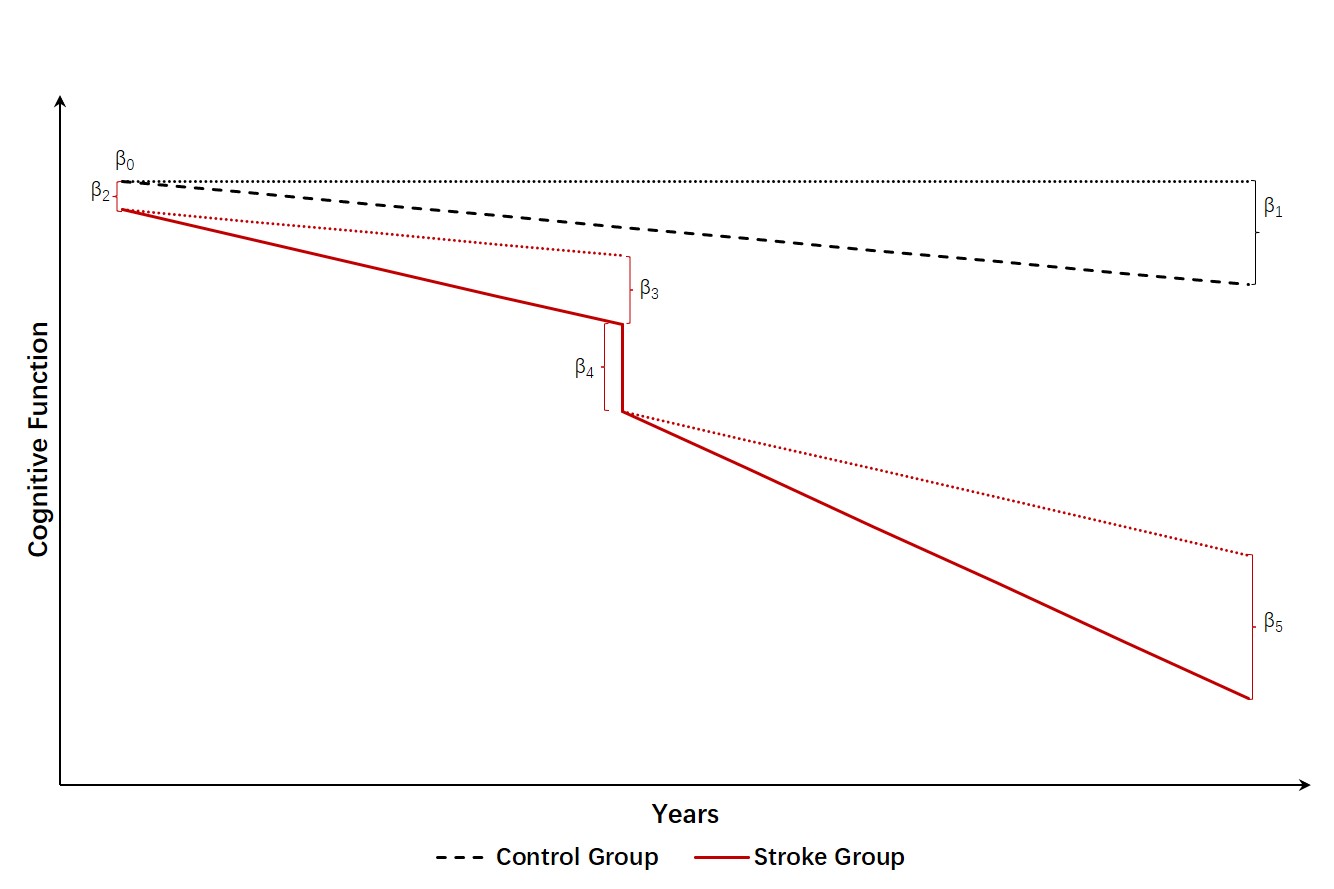
 The conceptual model of our study. Time on the x-axis is the years from the date of the first cognitive test. Y-axis is the cognitive function. The black dashed line represents the possible trajectory of the control group without incident stroke. We hypothesized their cognitive function declined annually due to aging. The cognitive trajectories of the stroke group (red lines) consisted of the trajectories before stroke, an acute decline at the time of stroke, and an accelerated decline after stroke.

$\beta$_0_: The predicted cognition of the without-stroke group at time t=0.

$\beta$_1_: The difference in cognition from time t to time t+1 among the without-stroke group. In other words, it’s the average slope of cognition of the entire without-stroke group.

$\beta$_2_: The difference in cognition at time t=0 in the stroke group compared to the without-stroke group. It was named ‘difference in baseline’ in Table 2.

$\beta$_3_: The difference in slope in the stroke group compared to the without-stroke group in the pre-stroke period.

$\beta$_4_: The ‘acute cognitive change’ at the stroke point among the stroke group, measured as the first post-stroke cognitive score minus the last pre-stroke cognitive score.

Let us hypothesize a person received cognitive tests in 2011, 2013, 2015, and 2018, and got a stroke in 2014. We define C_2011_ as the cognitive score in 2011. C_2015_ $-$ C_2013_ $=$ (C_2014_ $-$ C_2013_) $+$ (C_2015_ $-$ C_2014_) *we don’t know the cognitive score in 2014, since the score wasn’t recorded during this year* = annual decline rate before stroke + annual decline rate after stroke $=$ (C_2013_ $-$ C_2011_)^*^1/2 $+$ (C_2018_ $-$ C_2015_)^*^1/3. But, the actual C_2015_ $-$ C_2013_ is more than (C_2013_ $-$ C_2011_)^*^1/2 $+$ (C_2018_ $-$ C_2015_)^*^1/3, which means the cognitive scores declined more than expected. The decline exceeding expectation is the “acute cognitive decline”. This is in accordance with the phenomenon that the incidence rate of dementia within one year after stroke was 2 to 5 times higher than that one year after stroke.

$\beta$_5_: The change in slope in the post-stroke period compared to the pre-stroke period. After stroke, we hypothesized the cognitive decline rate was combined with the pre-stroke decline rate and an accelerated decline caused by stroke. We assumed stroke affects cognition in all years after stroke.

## Figure S2. Kaplan-Meier curves for the cumulative risk of stroke in tertiles. Kaplan-Meier curves, the Wilcoxon test, and the log-rank test were used to compare the cumulative risk of stroke over tertiles of cognitive scores. Group 1, the highest baseline cognitive tertile; Group 2, the mid baseline cognitive tertile; Group 3, the lowest baseline cognitive tertile. Panel a, global cognition z scores; Panel b, episodic memory; Panel c, TICS-10 test scores.


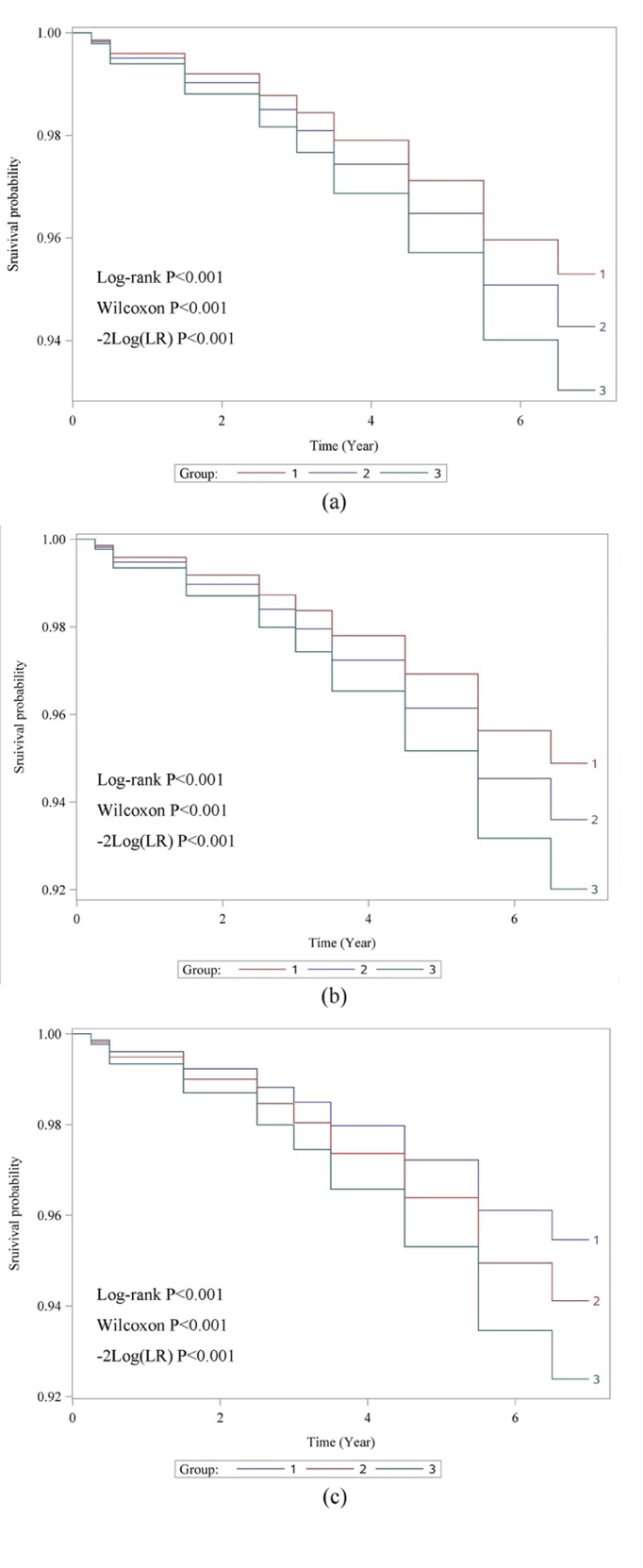


# Supplemental Tables

## Table S1. Age-standardised stroke incidence rates^a^

| Follow-up | Person-years | Number of incident stroke | Incident rate per 1000 person-years (95% CI) |
| --- | --- | --- | --- |
| 0-2 years | 1925.4 | 129 | 4.7 (1.6, 7.7) |
| 2-4 years | 2829.5 | 322 | 6.0 (3.5, 8.4) |
| 4-7 years | 7459.1 | 610 | 5.7 (4.1, 7.6) |

^a^Stroke incidence was analyzed for three study periods (2011-2013, 2013-2015, 2015-2018). Age-standardized rates per 1000 individuals were calculated by the direct method with the 2002 WHO world standard as the standard. Incidence rates per 1 000 population per year with corresponding 95% CIs were calculated by use of standard approaches.

## Table S2. Number of available cognition measurements in each wave

|  | Wave 1 | Wave 2 | Wave 3 | Wave 4 |
| --- | --- | --- | --- | --- |
| Without-stroke | 12701 (100%) | 11386 (89.7%) | 11023 (86.8%) | 8762 (69.0%) |
| Stroke | 610 (100%) | 557 (91.3%) | 550 (90.2%) | 458 (75.1%) |

## Table S3. Comparison of baseline characteristics between participants included (n=13311) and excluded due to loss to follow-up (n=1462)^a^

|  | Included | Excluded | P value^a^ |
| --- | --- | --- | --- |
|  | (n = 13311) | (n = 1462) |  |
| Continuous variables, mean (SD) |  | | |
| Age | 58.7 (9.2) | 64.3 (12.5) | <0.001 |
| Number of IADLs | 0.2 (0.6) | 0.6 (1.3) | <0.001 |
| Episodic memory | 3.3 (1.9) | 2.7 (2.1) | <0.001 |
| Visuospatial ability | 0.6 (0.5) | 0.6 (0.5) | 0.003 |
| TICS-10 test scores | 6.6 (2.9) | 6.0 (3.3) | <0.001 |
| Categorical variables, n (%) |  | | |
| Males | 6258 (47.0) | 664 (48.8) | 0.220 |
| Education |  |  | <0.001 |
| Illiterate | 3557 (26.7) | 447 (32.8) |  |
| Primary school | 5326 (40.0) | 258 (42.9) |  |
| Middle school | 2792 (21.0) | 235 (17.3) |  |
| High school and above | 1633 (12.3) | 228 (16.8) |  |
| Marital status |  |  | <0.001 |
| Married | 11812 (88.7) | 1061 (78.0) |  |
| Other status | 1499 (11.3) | 401 (22.0) |  |
| Residential area |  |  | <0.001 |
| Urban | 2813 (21.1) | 589 (43.3) |  |
| Rural | 10498 (78.9) | 772 (56.7) |  |
| Current smoking | 5156 (38.7) | 560 (41.8) | 0.085 |
| Current drinking | 3393 (25.5) | 288 (21.8) | <0.001 |
| Hypertension | 3010(22.6) | 399 (29.3) | <0.001 |
| Dyslipidemia | 1149 (8.7) | 125 (9.2) | 0.480 |
| Diabetes | 692 (5.2) | 97 (7.1) | 0.004 |
| Cancer | 116 (0.9) | 26 (1.9) | 0.001 |
| Lung Diseases | 1306 (9.8) | 168 (12.3) | 0.004 |
| Heart problems | 1510 (11.3) | 201 (14.8) | <0.001 |
| Depression | 2780 (20.9) | 311 (22.9) | 0.094 |

^a^Calculated using ANOVA for continuous covariates and χ^2^ test for categorical covariates.

TICS-10: 10-item Telephone Interview of Cognitive Status.

## Table S4. Adjusted changes in cognitive function over time: including 423 participants with a history of stroke at baseline (664 with incident stroke and 13070 without stroke during follow-up)^a^

|  | Global cognition | | | | |  | Episodic memory | | | | |  | Visuospatial ability | | | | |  | TICS-10 | | | | |
| --- | --- | --- | --- | --- | --- | --- | --- | --- | --- | --- | --- | --- | --- | --- | --- | --- | --- | --- | --- | --- | --- | --- | --- |
|  | Model A |  |  | Model B |  |  | Model A |  |  | Model B |  |  | Model A |  |  | Model B |  |  | Model A |  |  | Model B |  |
|  | β  (95% CI) | P |  | β  (95% CI) | P |  | β  (95% CI) | P |  | β  (95% CI) | P |  | β  (95% CI) | P |  | β  (95% CI) | P |  | β  (95% CI) | P |  | β  (95% CI) | P |
| Variables |  |  |  |  |  |  |  |  |  |  |  |  |  |  |  |  |  |  |  |  |  |  |  |
| Baseline age | -0.018  (-0.020, -0.017) | <0.001 |  | -0.018  (-0.020, -0.017) | <0.001 |  | -0.021  (-0.023, -0.020) | <0.001 |  | -0.022  (-0.023, -0.020) | <0.001 |  | -0.012  (-0.013, -0.011) | <0.001 |  | -0.012  (-0.013, -0.011) | <0.001 |  | -0.009  (-0.010, -0.007) | <0.001 |  | -0.009  (-0.010, -0.007) | <0.001 |
| Intercept of without-stroke group | 0.221  (0.114, 0.328) | <0.001 |  | 0.221  (0.115, 0.328) | <0.001 |  | 0.538  (0.434, 0.642) | <0.001 |  | 0.538  (0.433, 0.642) | <0.001 |  | 0.197  (0.092, 0.301) | <0.001 |  | 0.196  (0.092, 0.300) | <0.001 |  | 0.176  (0.075, 0.278) | <0.001 |  | 0.177  (0.075, 0.278) | <0.001 |
| Difference in baseline^b^ | 0.017  (-0.048, 0.082) | 0.606 |  | 0.017  (-0.050, 0.085) | 0.613 |  | 0.051  (-0.017, 0.119) | 0.142 |  | 0.041  (-0.030, 0.112) | 0.258 |  | 0.004  (-0.068, 0.076) | 0.909 |  | 0.27  (-0.049, 0.102) | 0.489 |  | 0.026  (-0.033, 0.085) | 0.389 |  | -0.006  (-0.068, 0.056) | 0.849 |
| Slope of without-stroke group | -0.032  (-0.035, -0.030) | <0.001 |  | -0.032  (-0.035, -0.030) | <0.001 |  | -0.014  (-0.017, -0.011) | <0.001 |  | -0.014  (-0.016, -0.012) | <0.001 |  | -0.038  (-0.041, -0.034) | <0.001 |  | -0.308  (-0.040, -0.034) | <0.001 |  | -0.050  (-0.052, -0.048) | <0.001 |  | -0.050  (-0.052, -0.048) | <0.001 |
| Difference in slope before stroke^c^ | -0.006  (-0.023, 0.011) | 0.507 |  | -0.006  (-0.026, 0.014) | 0.556 |  | -0.010  (-0.029, 0.009) | 0.295 |  | -0.005  (-0.027, 0.016) | 0.615 |  | -0.004  (-0.025, 0.017) | 0.706 |  | -0.015  (-0.039, 0.009) | 0.231 |  | -0.014  (-0.030, 0.001) | 0.076 |  | 0.002  (-0.016, 0.021) | 0.825 |
| Acute change after stroke^d^ | -0.138  (-0.227, -0.049) | 0.002 |  | -0.135  (-0.228, -0.042) | 0.004 |  | -0.124  (-0.218, -0.029) | 0.010 |  | -0.114  (-0.212, -0.015) | 0.024 |  | -0.094  (-0.203, -0.014) | 0.089 |  | -0.131  (-0.246, -0.015) | 0.027 |  | -0.053  (-0.138, 0.032) | 0.221 |  | -0.027  (-0.114, 0.059) | 0.534 |
| Changes in slope after stroke | None | None |  | -0.003  (-0.035, 0.029) | 0.857 |  | None | None |  | -0.012  (-0.043, 0.019) | 0.454 |  | None |  |  | 0.032  (-0.005, 0.092) | 0.088 |  | None |  |  | -0.041  (-0.067, -0.015) | 0.002 |
|  |  |  |  |  |  |  |  |  |  |  |  |  |  |  |  |  |  |  |  |  |  |  |  |
| Log likelihood | -47397.5 |  |  | - 47396.4 |  |  | - 58785.4 |  |  | - 58782.6 |  |  | -59977.7 |  |  | -59973.45 |  |  | -54718.2 |  |  | -54710.1 |  |

^*All coefficients and confidence intervals were shown in the form of z score.^

^a^Adjusted for baseline age (shown in Line “Baseline age”), sex, education, marital status, residential area, current smoking, current drinking, hypertension, dyslipidemia, diabetes, cancer, lung diseases, heart problems, depression, and number of IADLs.

^b^The difference in baseline cognitive scores in the stroke group compared to the without-stroke group.

^c^The difference in slope rate in the stroke group during pre-stroke period compared to the without-stroke group during the whole follow-up period.

^d^The amount the cognitive scores changed at the “stroke point” among the stroke group, measured as the first post-stroke value minus the last pre-stroke value.

## Table S5. Adjusted changes in cognitive function over time: only including participants received cognitive tests in all four waves (388 with incident stroke and 7394 without stroke during 7 years)^a^

|  | Global cognition | | | | |  | Episodic memory | | | | |  | Visuospatial ability | | | | |  | TICS-10 | | | | |
| --- | --- | --- | --- | --- | --- | --- | --- | --- | --- | --- | --- | --- | --- | --- | --- | --- | --- | --- | --- | --- | --- | --- | --- |
|  | Model A |  |  | Model B |  |  | Model A |  |  | Model B |  |  | Model A |  |  | Model B |  |  | Model A |  |  | Model B |  |
|  | β  (95% CI) | P |  | β  (95% CI) | P |  | β  (95% CI) | P |  | β  (95% CI) | P |  | β  (95% CI) | P |  | β  (95% CI) | P |  | β  (95% CI) | P |  | β  (95% CI) | P |
| Variables |  |  |  |  |  |  |  |  |  |  |  |  |  |  |  |  |  |  |  |  |  |  |  |
| Baseline age | -0.014  (-0.015, -0.012) | <0.001 |  | -0.014  (-0.015, -0.012) | <0.001 |  | -0.019  (-0.020, -0.017) | <0.001 |  | -0.019  (-0.021, -0.017) | <0.001 |  | -0.009  (-0.010, -0.007) | <0.001 |  | -0.009  (-0.010, -0.007) | <0.001 |  | -0.003  (-0.005, -0.001) | <0.001 |  | -0.003  (-0.005, -0.001) | <0.001 |
| Intercept of without-stroke group | 0.062  (-0.077, 0.200) | 0.384 |  | 0.062  (-0.076, 0.201) | 0.379 |  | 0.421  (0.286, 0.556) | <0.001 |  | 0.422  (0.287, 0.558) | <0.001 |  | 0.141  (0.004, 0.278) | 0.044 |  | 0.140  (0.003, 0.277) | 0.045 |  | -0.056  (-0.185, 0.074) | 0.398 |  | -0.055  (-0.184, 0.075) | 0.408 |
| Difference in baseline^b^ | -0.005  (-0.083, 0.072) | 0.894 |  | -0.012  (-0.094, 0.067) | 0.770 |  | 0.008  (-0.077, 0.094) | 0.846 |  | -0.009  (-0.099, 0.080) | 0.840 |  | -0.010  (-0.103, 0.083) | 0.831 |  | 0.017  (-0.080, 0.115) | 0.725 |  | 0.005  (-0.068, 0.077) | 0.903 |  | -0.028  (-0.104, 0.048) | 0.468 |
| Slope of without-stroke group | -0.032  (-0.035, -0.029) | <0.001 |  | -0.032  (-0.035, -0.029) | <0.001 |  | -0.003  (-0.006, 0.001) | 0.122 |  | -0.003  (-0.006, 0.004) | 0.121 |  | -0.038  (-0.043, -0.035) | <0.001 |  | -0.039  (-0.043, -0.035) | <0.001 |  | -0.037  (-0.040, -0.035) | <0.001 |  | -0.037  (-0.040, -0.035) | <0.001 |
| Difference in slope before stroke^c^ | -0.004  (-0.024, 0.016) | 0.707 |  | 0.000  (-0.024, 0.023) | 0.977 |  | -0.001  (-0.023, 0.022) | 0.963 |  | 0.008  (-0.018, 0.033) | 0.556 |  | -0.001  (-0.025, 0.028) | 0.936 |  | -0.011  (-0.041, 0.019) | 0.469 |  | -0.013  (-0.030, 0.0.006) | 0.175 |  | 0.003  (-0.018, 0.024) | 0.774 |
| Acute change after stroke^d^ | -0.132  (-0.236, -0.027) | 0.014 |  | -0.122  (-0.229, -0.015) | 0.025 |  | -0.170  (-0.284, -0.055) | 0.004 |  | -0.151  (-0.270, -0.032) | 0.013 |  | -0.130  (-0.268, 0.007) | 0.063 |  | -0.174  (-0.320, -0.029) | 0.019 |  | -0.006  (-0.104, 0.093) | 0.912 |  | 0.022  (-0.078, 0.122) | 0.660 |
| Changes in slope after stroke | None | None |  | -0.003  (-0.050, 0.024) | 0.489 |  | None | None |  | -0.025  (-0.063, 0.014) | 0.211 |  | None |  |  | 0.040  (-0.006, 0.087) | 0.091 |  | None |  |  | -0.043  (-0.073, -0.013) | 0.005 |
|  |  |  |  |  |  |  |  |  |  |  |  |  |  |  |  |  |  |  |  |  |  |  |  |
| Log likelihood | -31260.7 |  |  | -31258.6 |  |  | -35836.3 |  |  | -35834.8 |  |  | -38553.6 |  |  | -38550.5 |  |  | -30348.1 |  |  | -30343.1 |  |

^*All coefficients and confidence intervals were shown in the form of z score.^

^a^Adjusted for baseline age (shown in Line “Baseline age”), sex, education, marital status, residential area, current smoking, current drinking, hypertension, dyslipidemia, diabetes, cancer, lung diseases, heart problems, depression, and number of IADLs.

^b^The difference in baseline cognitive scores in the stroke group compared to the without-stroke group.

^c^The difference in slope rate in the stroke group during pre-stroke period compared to the without-stroke group during the whole follow-up period.

^d^The amount the cognitive scores changed at the “stroke point” among the stroke group, measured as the first post-stroke value minus the last pre-stroke value

## Table S6. Effect of living in urban areas on the effect of stroke on the following cognitive trajectories

| Variables | Global cognition | Episodic memory | Visuospatial ability | TICS-10 test |
| --- | --- | --- | --- | --- |
| Difference in baseline | 0.069  (-0.097, 0.236) | 0.020  (-0.173, 0.213) | 0.190  (-0.006, 0.385) | -0.036  (-0.213, 0.141) |
| Difference in slope before stroke | 0.006  (-0.043, 0.055) | 0.012  (-0.045, 0.070) | -0.029  (-0.091, 0.034) | 0.030  (-0.020, 0.081) |
| Acute change after stroke | -0.029  (-0.267, 0.210) | 0.036  (-0.248, 0.320) | -0.011  (-0.331, 0.310) | -0.065  (-0.323, 0.192) |
| Changes in slope after stroke | -0.012  (-0.092, 0.067) | 0.000  (-0.091, 0.090) | -0.014  (-0.116, 0.088) | -0.022  (-0.100, 0.056) |

All P$>$0.05.

All coefficients and confidence intervals are shown as z scores.

TICS-10: 10-item Telephone Interview of Cognitive Status.

## Table S7. Effect of education on the effect of stroke on the following cognitive trajectories

| Variables | Global cognition | Episodic memory | Visuospatial ability | TICS-10 test |
| --- | --- | --- | --- | --- |
| Primary school vs illiterate | | | | |
| Difference in baseline | 0.685  (0.510, 0.861) ^***^ | 0.236  (0.052, 0.420) ^*^ | 0.779  (0.571, 0.987) ^***^ | 0.613  (0.426, 0.800) ^***^ |
| Difference in slope before stroke | 0.001  (-0.048, 0.051) | 0.014  (-0.042, 0.070) | -0.042  (-0.105, 0.021) | 0.017  (-0.032, 0.067) |
| Acute change after stroke | -0.179  (-0.428, 0.070) | -0.072  (-0.341, 0.197) | -0.401  (-0.733, -0.068) ^*^ | 0.094  (-0.160, 0.349) |
| Changes in slope after stroke | 0.099  (0.010, 0.188) ^*^ | 0.102  (0.011, 0.192) ^*^ | 0.129  (0.021, 0.238) ^*^ | -0.028  (-0.106, 0.051) |
| High school and above vs illiterate | | | | |
| Difference in baseline | 0.668  (0.480, 0.856) ^***^ | 0.107  (-0.099, 0.313) | 0.815  (0.607, 1.023) ^***^ | 0.636  (0.440, 0.831) ^***^ |
| Difference in slope before stroke | 0.017  (-0.034, 0.067) | 0.061  (0.001, 0.121) ^*^ | -0.050  (-0.112, 0.013) | 0.019  (-0.034, 0.072) |
| Acute change after stroke | -0.258  (-0.506, -0.011) ^*^ | -0.195  (-0.494, 0.104) | -0.371  (-0.688, -0.054) ^*^ | -0.018  (-0.283, 0.247) |
| Changes in slope after stroke | 0.105  (0.021, 0.189) ^*^ | 0.131  (0.038, 0.225) ^**^ | 0.112  (0.007, 0.216) ^*^ | -0.019  (-0.104, 0.065) |

^*^P$<$0.05.

^**^P$<$0.01.

^***^P$<$0.001.

All coefficients and confidence intervals are shown as z scores.

TICS-10: 10-item Telephone Interview of Cognitive Status.

## Table S8. Effect of baseline cognition on the effect of stroke on the following cognitive trajectories

| Variables | Global cognition | Episodic memory | TICS-10 test |
| --- | --- | --- | --- |
| Median tertile vs highest tertile | | | |
| Difference in baseline | -0.664  (-0.795, -0.534) ^***^ | -0.750  (-0.900, -0.600) ^***^ | -0.916  (-1.051, -0.780) ^***^ |
| Difference in slope before stroke | 0.063  (0.017, 0.108) ^**^ | 0.069  (0.017, 0.122) ^**^ | 0.095  (0.049, 0.142) ^***^ |
| Acute change after stroke | -0.050  (-0.258, 0.158) | 0.134  (-0.112, 0.379) | 0.123  (-0.102, 0.347) |
| Changes in slope after stroke | -0.009  (-0.085, 0.066) | -0.079  (-0.164, 0.006) | -0.060  (-0.134, 0.013) |
| Lowest tertile vs highest tertile | | | |
| Difference in baseline | -1.025  (-1.200, -0.850) ^***^ | -1.370  (-1.571, -1.170) ^***^ | -1.517  (-1.716, -1.319) ^***^ |
| Difference in slope before stroke | 0.120  (0.072, 0.168) ^***^ | 0.196  (0.140, 0.252) ^***^ | 0.175  (0.123, 0.228) ^***^ |
| Acute change after stroke | 0.167  (-0.069, 0.402) | 0.016  (-0.264, 0.295) | 0.140  (-0.119, 0.400) |
| Changes in slope after stroke | -0.142  (-0.222, -0.062) ^***^ | -0.155  (-0.245, -0.065) ^***^ | -0.151  (-0.233, -0.069) ^***^ |

^*^P$<$0.05.

^**^P$<$0.01.

^***^P$<$0.001.

All coefficients and confidence intervals are shown as z scores.

TICS-10: 10-item Telephone Interview of Cognitive Status.

## Table S9. Effect of 1-year increase in baseline age on the effect of stroke on the following cognitive trajectories

| Variables | Global cognition | Episodic memory | Visuospatial ability | TICS-10 test |
| --- | --- | --- | --- | --- |
| Difference in baseline | 0.002  (-0.005, 0.010) | 0.011  (0.002, 0.019) ^*^ | -0.005  (-0.014, 0.004) | 0.001  (-0.007, 0.009) |
| Difference in slope before stroke | -0.001  (-0.003, 0.001) | -0.003  (-0.005, 0.000) | 0.001  (-0.002, 0.003) | -0.001  (-0.003, 0.002) |
| Acute change after stroke | 0.002  (-0.008, 0.013) | 0.001  (-0.011, 0.013) | 0.006  (-0.008, 0.020) | 0.001  (-0.010, 0.012) |
| Changes in slope after stroke | -0.001  (-0.005, 0.003) | 0.000  (-0.005, 0.004) | -0.001  (-0.006, 0.004) | 0.000  (-0.004, 0.003) |

^*^P$<$0.05.

All coefficients and confidence intervals are shown as z scores.

TICS-10: 10-item Telephone Interview of Cognitive Status.

## Table S10. Effect of female sex on the effect of stroke on the following cognitive trajectories

| Variables | Global cognition | Episodic memory | Visuospatial ability | TICS-10 test |
| --- | --- | --- | --- | --- |
| Difference in baseline | -0.021  (-0.154, 0.112) | 0.095  (-0.060, 0.249) | -0.118  (-0.274, 0.038) | -0.007  (-0.149, 0.135) |
| Difference in slope before stroke | 0.017  (-0.021, 0.056) | 0.000  (-0.046, 0.046) | 0.020  (-0.029, 0.069) | 0.020  (-0.021, 0.060) |
| Acute change after stroke | 0.171  (-0.015, 0.356) | 0.064  (-0.155, 0.282) | 0.288  (0.041, 0.534) ^*^ | -0.011  (-0.209, 0.187) |
| Changes in slope after stroke | -0.032  (-0.096, 0.031) | -0.030  (-0.101, 0.041) | -0.058  (-0.138, 0.022) | 0.010  (-0.051, 0.071) |

^*^P$<$0.05.

All coefficients and confidence intervals are shown as z scores.

TICS-10: 10-item Telephone Interview of Cognitive Status.

## Table S11. Effect of western stroke medicine on the effect of stroke on the following cognitive trajectories

| Variables | Global cognition | Episodic memory | Visuospatial ability | TICS-10 test |
| --- | --- | --- | --- | --- |
| Difference in baseline | 0.011  (-0.087, 0.110) | 0.063  (-0.052, 0.178) | 0.020  (-0.096, 0.136) | -0.056  (-0.162, 0.049) |
| Difference in slope before stroke | -0.017  (-0.042, 0.008) | -0.033  (-0.063, -0.003) ^*^ | -0.006  (-0.039, 0.026) | 0.003  (-0.022, 0.029) |
| Acute change after stroke | -0.265  (-0.453, -0.077) ^*^ | -0.093  (-0.315, 0.129) | -0.440  (-0.689, -0.191) ^***^ | -0.202  (-0.398, -0.005) ^*^ |
| Changes in slope after stroke | 0.097  (0.024, 0.170) ^*^ | 0.074  (-0.010, 0.158) | 0.169  (0.076, 0.261) ^***^ | 0.027  (-0.044, 0.098) |

^*^P$<$0.05.

^**^P$<$0.01.

^**^P$<$0.001.

All coefficients and confidence intervals are shown as z scores.

TICS-10: 10-item Telephone Interview of Cognitive Status.

## Table S12. Association between baseline cognitive function and new-onset stroke

| New-onset stroke | Group 1^a^ | Group 2 | Group 3 |
| --- | --- | --- | --- |
| Global cognition |  |  |  |
| Cases n (%) | 187 (4.0%) | 222 (4.7%) | 204 (5.0%) |
| Model 0 | 1 (Ref.) | 1.221 (1.005, 1.483)^b^ | 1.228 (1.112, 1.357) |
| Model 1 | 1 (Ref.) | 1.133 (0.920, 1.394) | 1.058 (0.927, 1.207) |
| Episodic memory |  |  |  |
| Cases n (%) | 229 (4.1%) | 189 (5.0%) | 195 (5.1%) |
| Model 0 | 1 (Ref.) | 1.348 (1.112, 1.635) | 1.259 (1.144, 1.386) |
| Model 1 | 1 (Ref.) | 1.126 (0.921, 1.376) | 1.096 (0.985, 1.128) |
| TICS-10 test |  |  |  |
| Cases n (%) | 182 (3.8%) | 258 (4.8%) | 173 (5.3%) |
| Model 0 | 1 (Ref.) | 1.321 (1.093, 1.597) | 1.309 (1.179, 1.453) |
| Model 1 | 1 (Ref.) | 1.252 (1.025, 1.530) | 1.137 (0.990, 1.305) |

^a^Group 1, the highest baseline cognitive tertile; Group 2, the mid baseline cognitive tertile; Group 3, the lowest baseline cognitive tertile.

^b^Hazard ratios (95% CI), for all such values.

Model 0: unadjusted.

Model 1: Adjusted for baseline age, sex, education, marital status, residential area, current smoking, current drinking, hypertension, dyslipidaemia, diabetes, cancer, lung diseases, heart problems, depression, and number of IADLs.
